# Editorial: Global excellence in cardiovascular medicine: Asia and Australasia

**DOI:** 10.3389/fcvm.2025.1645942

**Published:** 2025-07-24

**Authors:** Zhonghua Sun

**Affiliations:** ^1^ Curtin Medical School, Curtin University, Perth, WA, Australia; ^2^ Curtin Medical Research Institute (Curtin MRI), Curtin University, Perth, WA, Australia

**Keywords:** cardiovascular disease, diagnosis, risk factor, prevention, management

**Editorial on the Research Topic**
Global excellence in cardiovascular medicine: Asia and Australasia

Frontiers in Cardiovascular Medicine has introduced the idea of organising a series of special edition research topics aiming to highlight the latest advancements of cardiovascular research across the globe. This Research Topic section on “*Global excellence in cardiovascular medicine: Asia and Australasia*” aims to attract researchers from Asia and Australasia regions to submit high-quality research studies in the field of cardiovascular disease.

A special Research Topic features 11 manuscripts from global researchers on innovative studies in cardiovascular medicine, shedding light on the recent progress across the entire breadth of cardiovascular disease (CVD), and addressing future challenges in cardiovascular disease research.

More than half of these studies (6 out of 11) focused on clinical cardiology and cardiovascular risk. Dwiputra et al. used the SMART-REACH model to estimate the risk of recurrent cardiovascular event among 3,209 Indonesian patients diagnosed with atherosclerotic CVD in terms of 10-year and lifetime risk of developing major cardiovascular events and non-cardiovascular mortality. They reported that an average 10-year risk of recurrent cardiovascular events was 30.2%, and a lifetime risk was 62.5%. Females exhibited a higher average risk than males, while patients with diabetes mellitus were found to have the highest average risk for both 10-year and lifetime events compared to those with active smokers and hypertension.

The importance of using white blood cell (WBC) count as an independent predictor for risk assessment of CVD was highlighted in a study by Jiang et al. Authors measured WBC counts at baseline from 1,933 Asian population from the STABILITY randomized controlled trial to determine the role of WBC in predicting the cardiovascular disease risk in patients with stable coronary artery disease. Their results showed that patients with the lowest WBC were associated with significantly lower incidences of primary and secondary cardiovascular events than patients with higher WBC counts by 4 years of follow-up.

Zhou et al. investigated the application of novel inflammatory biomarkers in cardiovascular surgeries involving cardiopulmonary bypass (CPB). Of 332 patients enrolled in the study, 96 were allocated to the CPB group and 236 to the non-CPB group. After propensity score matching, 58 patients were included in both groups. A consistent increase of novel inflammatory biomarkers (neutrophil/lymphocyte ratio-NLR, platelet/lymphocyte ratio-PLR, systemic inflammation index-SII, pan-immune inflammatory value-PIV) was found after cardiovascular surgeries involving CPB, reaching the peak values on the first day after surgery, with NLR considered a reliable prognostic indicator.

Cong et al. enrolled 30 children (mean age 3.41 ± 2.29 years) with successful perimembranous ventricular septal defect (Pm-VSD) closure (a novel fully biodegradable implantable device) with a follow-up period of 3 years. Outcome variables included 1-year, 2-year, and 3-year follow-up after device implantation. Furthermore, 30 healthy children with matched age and sex were included as a control group to compare myocardial function at the end of the 3rd year. All 30 cases completed the 1-year follow-up, 24 completed the 2-year follow-up and 13 completed the all three follow-ups. The size of the novel medical devices gradually decreased over time and were eventually invisible when imaged with echocardiography in the 3rd year. When compared to the control group, there were no significant differences in the myocardial deformation parameters (*p* > 0.05), highlighting the use of novel fully biodegradable occluder for VSD.

Nguyen et al. used speckle tracking echocardiography to study the association of dimensional and strain changes in left ventricle (LV) and left atrium (LA) with different stages of chronic kidney disease (CKD) in 169 patients. They found a positive association between CKD severity and LV and LA diameter changes, with significantly increased in LV and LA volumes in patients with severe CKD (*p* < 0.001). A negative correlation was found between the LV global longitudinal strain and CKD severity, with progressive reduction in LV and LA strain and left ventricular ejection fraction when CKD progresses to more severe (*p* < 0.05).

Kaneta et al. assessed the burden of prostate diseases (benign prostate hyperplasia and prostate cancer) and their association with prevalence and temporal trends of CVD in hospitalised patients with confirmed CVD. Of 6,078,487 patients analysed, the prevalence of prostate diseases was 5.7%, and it was significantly increased as the age increased. The lowest prevalence rates of prostate diseases were found in patients with acute coronary syndrome (4.5%), whereas the highest prevalence rates were observed in patients with heart failure (8.6%). This study emphasises the relationship between CVD and risk of developing prostate diseases.

Three studies presented researchers' different experiences involving development of coronary CT protocols, animal study of comparing two cardioplegia solutions and a systematic review and meta-analysis of dyslipidemia epidemiology in China. Kim et al. explored the feasibility of using coronary-aorta computed tomography (CACT) to evaluate the coronary arteries and aorta simultaneously as a dedicated protocol in 479 patients when compared to conventional aorta CT in 693 patients with aortic aneurysm. During the 3-year follow-up, a significantly higher incidence of coronary revascularization was observed in the CACT group than in the aorta CT group (11.2% vs. 4.0%, *p* < 0.001). The CACT group had significantly lower incidence of all-cause death or myocardial infarction than the aorta CT group (5.7% vs. 9.5%, *p* = 0.028). CACT is preferable over aorta CT in evaluating aortic aneurysm.

In their animal study, Yu et al. compared the outcomes after cardioplegia surgery by randomly allocating 12 male pigs to either receiving their in-house formation (Huaxi-1) or the commercial one (histidine-tryptophan-ketoglutarate, HTK). Use of Huaxin-1 solution led to higher concentrations of high-energy phosphates in myocardium, more rapid recovery of cardiac function and less injury to myocardium than the use of HTK solution.

Xia et al. conducted a systematic review and meta-analysis of 41 original studies summarized the prevalence, rates of awareness, control and treatment of dyslipidemia among Chinese adults. The pooled prevalence of dyslipidemia was 42.1%, with males having higher prevalence then females (47.3% vs. 38.8%). The pooled rates of awareness, treatment and control of dyslipidemia were 18.2%, 11.6% and 5.4%, respectively, with higher rates observed in females than males. Significantly higher rates of awareness, treatment and control were observed in urban residents than their rural counterparts (*p* < 0.001).

The remaining two studies reported rare cases of cardiac metastasis and primary cardiac tumour. Akabane et al. reported a rare case of cardiac metastasis of thyroid cancer in a 68-year-old woman presenting with acute arterial occlusion of right lower extremity and left atrial invasion due to pulmonary metastases from thyroid cancer. Kassab et al. reported another rare case of primary cardiac lymphoma in a 64-year-old man who presented the symptoms of superior vena cava syndrome to the emergent department.

We hope these articles dealing with various aspects of cardiovascular disease through analysis of a variety of risk factors or parameters, as well as utilising different technologies will help researchers to improve their understanding of the research activities across these regions, and guide future works through this special topic collection.

